# Inhibition of Biogenic Amine Production in Tyrosine Decarboxylase Broth Using Supercritical CO_2_ Extracts of Oregano, Cumin, Black Pepper, and Red Pepper

**DOI:** 10.1002/fsn3.72085

**Published:** 2026-07-05

**Authors:** Mustafa Hamza Mawlood Al Bayati, Mehmet Fatih Cengiz, Mariem Bouali, Pinar Yerlikaya

**Affiliations:** ^1^ Department of Agricultural Biotechnology, Faculty of Agriculture Akdeniz University Antalya Turkiye; ^2^ Department of Animal Sciences, College of Agricultural Engineering Sciences University of Sulaimani Sulaimani Iraq; ^3^ Department of Seafood Processing Technology, Faculty of Fisheries Akdeniz University Antalya Turkiye

**Keywords:** biogenic amines, decarboxylation, plant extracts, supercritical fluid carbon dioxide, tyrosine

## Abstract

In this study, the parameters of the supercritical fluid carbon dioxide (SCF‐CO_2_) extraction system were optimized to obtain the most suitable oregano, cumin, black pepper and red pepper plant extracts and the effects of the obtained extracts on the formation of biogenic amines (BA) in tyrosine decarboxylase medium (TDB) containing different species of microorganisms ( *Enterococcus faecalis* , *Escherichia coli* , *Klebsiella pneumoniae* , *Pseudomonas aeruginosa* , and *Staphylococcus aureus* ) were evaluated. BAs levels and active ingredients of the extracts (carvacrol, cumin‐aldehyde, piperin, and capsaicin) were determined by a validated HPLC method. According to the results, oregano and cumin SCF‐CO_2_ extracts at 0.50% completely inhibited the growth of all microorganisms tested in TDB. *E. faecalis* caused the highest total BA production (1153.88 mg/L) in TDB, followed by *K. pneumoniae* (677.78 mg/L) and *E. coli* (662.66 mg/L). Oregano extract (0.50%) reduced total biogenic amines produced by *E. faecalis* from 1153.88 to 124.14 mg/L, while cumin extract (0.50%) reduced total biogenic amines produced by *E. coli* from 662.66 to 267.14 mg/L. The extracts with the strongest effect on total BAs produced by *Enterococcus faecalis* , *Escherichia coli* , *Klebsiella pneumoniae* , *Pseudomonas aeruginosa* , and *Staphylococcus aureus* in TDB were determined to have the following concentrations: oregano (0.50%), cumin (0.50%), cumin (0.50%), black pepper (0.10%), and cumin (0.50%), respectively.

## Graphical abstract

The ability of supercritical CO_2_ extracts obtained from oregano, cumin, black pepper, and red pepper to inhibit biogenic amine formation by foodborne bacteria was evaluated in tryptone decarboxylase broth (TDB). Oregano and cumin extracts exhibited the strongest inhibitory activity, significantly reducing both bacterial growth and biogenic amine accumulation.

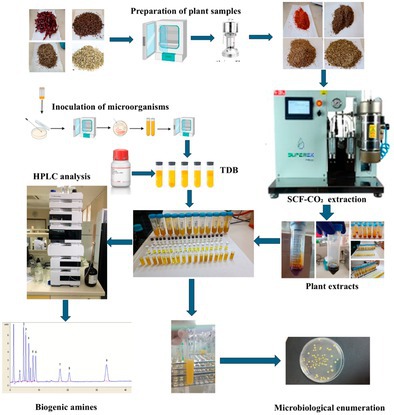

## 1 Introduction

Biogenic amines (BAs) are biologically active and nitrogenous organic compounds with low molecular weight, characterized by their aliphatic, aromatic, and heterocyclic structures (Erdag et al. 2018). These compounds are produced by removing the carboxyl group from amino acids by the activity of bacterial enzymes (particularly during microbial proteolysis) and are found naturally in plants, animals, and microorganisms (Al Bayatı and Cengiz 2024; Bita and Sharifian 2024; Wójcik et al. 2021; Zhang et al. 2024). The importance of BAs is reported as having the potential to cause health problems such as hypertension, allergic reactions, gastritis, headache, edema, depression and muscle pain, and particularly affecting the nervous, digestive, respiratory and cardiovascular systems. Therefore, they hold a critical role in public health and food safety (Ruiz‐Capillas and Herrero 2019). BAs are primarily formed in foods through the decarboxylation of specific amino acid precursors by microbial or endogenous decarboxylase enzymes under favorable conditions (Özoğul 2004). Amino acids commonly involved in biogenic amine formation include tyrosine, histidine, ornithine, phenylalanine, lysine, tryptophan, and arginine, which produce monoamines such as histamine (HIS), tyramine (TYR), tryptamine (TRP), and phenylethylamine (PHEA), as well as diamines such as putrescine (PUT) and cadaverine (CAD) (Gao et al. 2023).

The type and concentration of BAs found in foods varies with the microorganisms capable of producing decarboxylase enzymes, the amino acid concentration in the environment, and the availability of appropriate cofactors or inducers required for decarboxylase enzyme synthesis (Li and Lu 2020).

Bacterial species such as Escherichia, Salmonella, Pseudomonas, Streptococcus, Leuconostoc, Lactobacillus, Bacillus, Pediococcus, Citrobacter, Photobacterium, Klebsiella, Proteus, Shigella, and Clostridium have been reported in the literature to have the ability to produce BAs (Jeon et al. 2018). Therefore, BAs formation can be kept under control by identifying and regulating the critical factors affecting microbial growth and activity. The type and concentration of BAs produced depend on several factors such as microbial load, temperature, free amino acids, salt concentration, pH, and humidity (Saewa et al. 2021).

BA formation in food systems can be prevented by inhibiting microbial proliferation, mainly through freezing, refrigeration, radiation, hydrostatic pressure, modified atmosphere packaging, and food additives. The key to reducing BA formation in food products is to inhibit or prevent the growth of BA‐producing bacteria (Naila et al. 2010).

Additionally, although new approaches such as nitrite/nitrate chemicals and gamma radiation have been established for bacterial inactivation, concerns about increasing microbial resistance and potential residual risks of these systems have accelerated innovations in biocidal and therapeutic approaches. The main objective of these strategies is to effectively control target microorganisms while preserving food quality and ensuring consumer safety. Recent research has focused on plant extracts with secondary metabolite composition with inhibitory activity against bacteria, yeasts, and molds (Dai et al. 2025; de Castro et al. 2019; Rasheed et al. 2024). Scientific studies have revealed that the active substances and secondary metabolites found in some plants are effective even against resistant bacteria (El Asbahani et al. 2015; Silva et al. 2021). The antimicrobial properties of herbal extracts are primarily based on the phenolic compounds and other active elements found in their structures. These compounds interfere with the cell walls and membranes of bacteria, altering the permeability of the cells, leading to changes in the ion balance in the cytoplasm and interference with genetic material or energy metabolism, ultimately killing the cell (Chan et al. 2018).

SCF‐CO_2_, one of the mentioned extraction techniques, is a new and environmentally friendly extraction technique and has recently become the focus of attention due to its advantages such as residue‐free processing, affordable cost, higher purity of active ingredients and shorter extraction time (Al Bayati et al. 2024). The main purpose of this extraction process is to obtain the active ingredients in the plant material in high yield and purity, free from residues of solvents and auxiliary substances used in the process. Due to the mentioned advantages, SCF‐CO_2_ extraction is widely used in the food, cosmetic and pharmaceutical industries today due to its capacity to extract active ingredients from plant materials with high efficiency (Silva et al. 2021). The process is based on the principle of dissolving the active ingredients found in plant compounds in the supercritical liquid phase and isolating the active ingredients by exposing the solution to low pressure (Uwineza and Waśkiewicz 2020).

SCF‐CO_2_ is generally performed at low temperatures, which prevents structural damage to heat‐sensitive molecules. This process minimizes the chemical transformation and loss of active ingredients seen in other traditional extraction processes (López‐Hortas et al. 2022).

The primary objective of this study was to investigate how plant extracts obtained using the SCF‐CO_2_ extraction technique affect BAs levels in vitro. For this purpose, various plant extracts were obtained by SCF‐CO_2_ and tested at different concentrations in decarboxylation environments to determine which type and dosage were most effective in reducing BAs formation.

Although the antimicrobial properties of spice extracts and their effects on biogenic amine (BA) formation have been widely studied, little is known about the comparative efficacy of supercritical CO_2_ (SCF‐CO_2_) extracts obtained under optimized extraction conditions against BA‐producing foodborne pathogens. Moreover, previous research has predominantly focused on conventional solvent extracts, while the role of SCF‐CO_2_ extracts enriched in bioactive compounds such as carvacrol, cuminaldehyde, piperine, and capsaicin has received limited attention. To address this gap, the present study investigated the effects of optimized SCF‐CO_2_ plant extracts on BA production by different foodborne pathogenic bacteria in tyrosine decarboxylase broth and identified the most effective extract types and concentrations for suppressing BA formation. This approach provides new evidence linking optimized SCF‐CO_2_ extraction, antimicrobial activity, and the inhibition of BA accumulation in vitro.

## 2 Materials and Methods

### 2.1 Materials

The oregano ( *Origanum onites* ), cumin ( *Cuminum cyminum* ), black pepper ( *Piper nigrum* ), and red pepper ( *Capsicum annuum* L.), used in the study, were obtained from a supplier company operating in Antalya/Turkiye. In the statement made by the company, it was stated that the materials originated from Denizli/Türkiye, Burdur/Türkiye, Vietnam and India, respectively.

Five different foodborne bacteria, including *Enterococcus faecalis* ( *E. faecalis* , 20,212 ATCC), *Escherichia coli* ( *E. coli* , 25,921 ATCC), *Klebsiella pneumoniae* ( *K. pneumoniae* , 7,00,603 ATCC), *Pseudomonas aeruginosa* ( *P. aeruginosa* , 23,853 ATCC), and *Staphylococcus aureus* ( *S. aureus* , 29,213 ATCC) were used in the study. BAs analytical standards (dopamine (DOP), Tryptamine (TRP), cadaverine (CAD), putrescine (PUT), spermidine (SPD), spermine (SPR), phenylethylamine (PHEN), histamine (HIS), tyramine (TYR)) were purchased from Sigma Aldrich (St Louis, MO, USA).

### 2.2 Preparation of Plant Materials

The plant materials used in the study were dried in an oven at 35°C for approximately 48 h until the moisture content dropped below 1%. The content of moisture in the samples was analyzed by using the dry matter analysis protocol. After the drying process, the samples were ground by using a laboratory type mill (IKA MF 10 Basic, Staufen, Germany). The ground samples were passed through a 100 mesh sieve to obtain trial materials with homogeneous particle sizes.

### 2.3 Optimization of Extraction Conditions and Preparation of Plant Extracts

The extract containing active compounds obtained from plants was produced with SCF‐CO_2_. Plant materials were weighed 50 ± 1 g and placed into the reactor of the SCF‐CO_2_ extraction system. The parameters of the system (extraction pressure and temperature) were optimized by response surface methodology for each plant materials to obtain maximum amount of bioactive ingredients. According to results of our previous study, the extraction conditions varied depending on the nature of the raw material. For oregano and black pepper, optimal extraction was achieved at 30°C and 172 bar. In contrast, cumin and red pepper required higher temperatures or pressures, cumin was extracted at 52°C and 234 bar, and red pepper at 52°C and 172 bar. Under these conditions, 44.23% of carvacrol concentration for oregano, 0.72% of cumin aldehyde concentration for cumin, 61.10% of piperine concentration for black pepper and 5.29% of capsaicin concentration for red pepper were achieved. The extraction conditions reported above were selected based on a separate response surface methodology (RSM) optimization study conducted by our research group. The objective of that study was to maximize the recovery of the major bioactive compounds in each plant material. Since complete optimization models, response surface plots, and statistical parameters are part of an independent manuscript currently under review for publication, only the optimal extraction conditions and corresponding bioactive compound concentrations used in the present study are presented.

### 2.4 Determination of Active Compounds in the Extracts

The active compounds in each extract composition obtained under different SCF‐CO_2_ extraction conditions were analyzed. Separation was achieved on a C18 reversed‐phase column with dimensions of 5 mm × 150 mm and a particle size of 5 μm. The mobile phase consisted of acetonitrile (80%) and 1% acetic acid in ultra‐pure water (20%) (v/v), delivered at a constant flow rate of 1.0 mL/min, and the following wavelengths were used: 277 nm for Carvacrol, 260 nm for Cuminaldehyde, 280 nm for Capsaicin, and 343 nm for Piperine. The injection volume was set to 20 μL for all samples. Prior to sample analysis, the HPLC system was calibrated using analytical standards of active compounds (Carvacrol, Cuminaldehyde, Capsaicin, and Piperine). The standard stock solutions for each active compound were prepared individually by dissolving the respective standards in ethanol.

### 2.5 Preparation of Decarboxylase Broth

Decarboxylase broth was formulated according to the procedure developed by (Klausen and Huss 1987), to determine the types and levels of BAs produced in the presence of tyrosine and microbial strains. The Tyrosine Decarboxylase Broth (TDB) was prepared by dissolving 1 g of peptone, 0.5 g of Lab‐Lemco, 2.5 g of sodium chloride (NaCl), and 2.5 mg of pyridoxal‐HCl in 500 mL of distilled water, followed by the addition of 4.01 g of L‐tyrosine amino acid, and finally pH adjustment to 6.5. Portions of 10 mL were then dispensed into test tubes and sterilized in an autoclave (Tomy, SX 700E, Tokyo, Japan) at 121°C for 15 min.

### 2.6 Inoculation of Microorganisms and Plant Extracts Into TDB

Microorganisms known to contribute to BAs production ( *E. faecalis* , *E. coli* , *K. pneumoniae* , *P. aeruginosa* , and *S. aureus* ) grew individually in Nutrient Broth until they reached 10^8^ CFU/mL. 0.5 mL of each culture was transferred to aseptically sterilized TDB tubes. These tubes were incubated at 37°C for 72 h. To evaluate the effectiveness of plant extracts in reducing BAs formation, the extracts were prepared at three concentrations (0.02%, 0.10%, and 0.50%). Prepared extracts were added to TDB inoculated with selected microorganisms. The mixtures were then incubated for 72 h at 37°C. After the incubation period, the types and concentrations of BAs present in the broth were quantified using HPLC. An uninoculated TDB tube without bacterial inoculation or plant extract addition was included as a negative control and analyzed under the same experimental conditions. No detectable BA levels were observed in the uninoculated negative control, confirming that TDB itself did not contribute to BA formation. For this purpose, 5 mL of the upper phase of the solution was filtered by using a 0.22 μm filter and analyzed for BAs content. In addition to the BAs analysis microbiological analyses were also performed in the TDB solution. For this purpose, 0.1 mL of sample was taken from the developed bacterial culture and appropriate dilutions (up to 10^−10^) were prepared and the sample was inoculated onto plate count agar. Petri dishes were incubated for 72 h at 37°C, and the microorganisms growing on the Petri dish were counted.

### 2.7 Biogenic Amine Analysis

Types and levels of BAs in the TDB were analyzed using the benzoylation derivatization method reported by (Jia et al. 2013) with slight modifications. Briefly, 200 μL of samples or BAs standard solution were mixed with 500 μL of sodium‐borate buffer (0.2 M, pH 10.0) and 50 μL of Benzoyl chloride (Ben‐Cl at 3% in acetonitrile, v/v) in a vial (10 mL glass vial) with a screw cap. The mixture was mixed using a vortex for 30 s, followed by incubation in an ultrasonic water bath at 30°C for 30 min. 1.25 mL of saturated NaCl solution was added to stop the reaction. The benzoylated BAs were then extracted by adding 5 mL of diethyl ether, and then, from the upper organic phase, 3 mL was transferred to a tube and evaporated to dryness under a nitrogen gas stream. The dried residue was dissolved in 1 mL of methanol and subjected to HPLC. The analyses were performed using an Agilent 1200 HPLC system equipped with a diode array detector (DAD) set at a detection wavelength of 254 nm. Separation was achieved on a C18 reversed‐phase column with dimensions of 5 mm × 150 mm and a particle size of 5 μm. The mobile phase consisted of 55% methanol and 45% ultra‐pure water (v/v), delivered at a constant flow rate of 1.0 mL/min. The injection volume was set to 20 μL for all samples. Prior to sample analysis, the HPLC system was calibrated using analytical standards of BAs (DOP, PUT, CAD, TRP, PHEA, SPD, SPR, HIS, TYR). Standard stock solutions for each BA were prepared separately by dissolving the respective standards in 0.1 M HCl. These solutions were stored at +4°C in the dark until analysis. Each prepared standard solution was introduced into the HPLC system, and the retention times and peak intensities were recorded. To avoid retention time overlaps and interactions during the chromatographic analysis of individual analytical standards, method parameters (mobile phase composition, column temperature, flow rate and detector wavelength) were optimized. Using optimal conditions, the method was validated by reinjecting a series of BA standard mixtures into the HPLC system at concentrations of 0.2, 0.4, 1, 2, 4, 8, 10, and 20 mg/L for each BA.

### 2.8 Method Validation

Before determining the types and levels of BAs formed in the resulting solutions and the active compounds in plant extracts, the relevant chromatographic method was validated based on the parameters described below:

#### 2.8.1 Precision

In these studies, the repeatability of concentration values and the precision of retention times obtained from injections with standard substances and samples were evaluated. The relative standard deviation (RSD) of the results was used to determine the repeatability precision (Taverniers et al. 2004).

#### 2.8.2 Selectivity

The purpose of the selectivity test was to determine whether the analyte could be accurately measured in the presence of matrix. For this purpose, blank, analyte, sample, and matrix sample enriched with analyte injections were performed using the HPLC system, and obtained chromatograms were evaluated.

#### 2.8.3 Limit of Detection (LOD) and Limit of Quantification (LOQ)

Each of the lowest concentration standards from the calibration curve was injected 10 times into the HPLC instrument. The standard deviation of the peak areas obtained from these repeated injections was then calculated. Using the method outlined (Taverniers et al. 2004), the limit of quantification (LOQ) was calculated as 10 times the standard deviation, and the limit of detection (LOD) was calculated as three times the standard deviation.

#### 2.8.4 Linearity

Standard solutions of target analytes were prepared at least six different concentration levels, and each concentration was analyzed in triplicate. The resulting chromatographic data were processed using the HPLC system's ChemStation software to generate calibration curves. To ensure the reliability of the findings, the data were further analyzed using Minitab statistical software for validation purposes.

### 2.9 Statistical Analysis

In this study, statistical analyses were performed using the XLSTAT program. The impact of the extracts on BAs formation by bacteria was evaluated using analysis of variance (ANOVA). In the design, each variable was examined at three levels, and three repetitions were performed at the center point. The Duncan Multiple Range test was used to compare means. Statistical significance was established at *p* < 0.05, and the results were interpreted at a 95% confidence level.

## 3 Results and Discussion

### 3.1 Optimization of HPLC Method Parameters for Determination of BAs Levels

**FIGURE 1:**
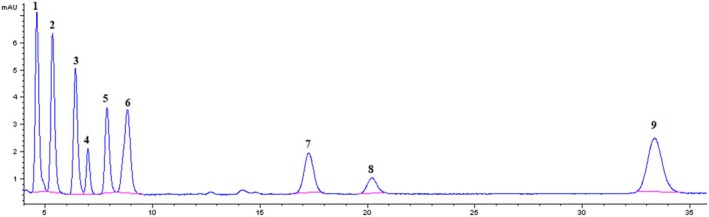
Analytical standard for biogenic amines (20 mg/L): DOP: 1, PUT: 2, CAD: 3, TRP: 4, PHEA: 5, SPD: 6, SPR: 7, HIS: 8, TYR: 9.

In the method validation study carried out to determine the types and levels of BA compounds, calibration curves were first created using BAs at different concentrations (DOP, PUT, CAD, TRP, PHEA, SPM, SPR, HIS, TYR). According to the results, the method was found to be linear (*r* ^*2*^ = > 0.996) within the specified concentration ranges (0.2–20 mg/L for biogenic amines). In addition, the findings obtained from the validation studies conducted on BAs standards are presented in Table 1, and the sample chromatogram obtained from the HPLC analyses performed during the study is presented in Figure 1. The findings obtained from the HPLC method validation study carried out within the scope of BA analysis show that the method provides high accuracy and reliability in determining the types and levels of BAs in the samples.

**TABLE 1:** Validation results for HPLC analysis of biogenic amines.

BAs	Linear range (ppm)	Calibration equation	Retention time (min)	RSD %	LOQ (ppm)	LOD (ppm)	*r* ^2^
DOP	0.62–20	*y* = 0.723 *x* − 0.631	02.62	1.771	0.845	0.254	0.998
PUT	0.15–20	*y* = 0.194 *x* + 0.374	04.83	3.236	1.332	0.400	0.999
CAD	0.15–20	*y* = 0.218 *x* + 0.183	05.63	3.593	1.463	0.439	0.999
TRP	0.31–20	*y* = 0.247 *x* + 0.221	06.84	3.412	1.376	0.413	0.999
PHEA	0.31–20	*y* = 0.317 *x* + 0.343	08.38	5.202	2.079	0.624	0.999
SPM	0.31–20	*y* = 0.273 *x* + 0.409	09.67	9.990	8.294	2.488	0.999
SPR	0.13–20	*y* = 0.321 *x* + 0.315	19.73	8.998	3.254	0.976	0.996
HIS	0.62–20	*y* = 0.503 *x* + 1.768	22.75	5.578	2.071	0.621	0.998
TYR	0.15–20	*y* = 0.146 *x* + 0.336	37.90	3.513	1.359	0.408	0.998

### 3.2 Effects of Extracts on Bacterial Growth in TDB

The ability of four different plant extracts to inhibit the growth of pathogenic strains ( *E. faecalis* , *E. coli* , *K. pneumoniae* , *P. aeruginosa* , and *S. aureus* ) at different concentrations is shown in Table 2. All bacterial strains were completely inhibited at a concentration of 0.50% oregano and cumin extract in TDB. In addition, it was observed that oregano and cumin extract significantly reduced the log CFU counts for *E. faecalis* and *E. coli* , especially at 0.10% concentration. The amounts of *E. faecalis* were detected as 2.45 and 3.76 log CFU/mL, respectively, and for *E. coli* , as 2.76 and 3.48 log CFU/mL. Oregano and cumin also showed broad efficacy against *K. pneumoniae* at all concentrations. *K. pneumoniae* and *P. aeruginosa* showed some resistance to the extracts; however, the number of CFUs was found to be reduced even at low concentrations of the extracts. *S. aureus* levels decreased continuously with increasing concentrations of all extracts. In general, oregano and cumin extracts were found to be prominent for their strong inhibitory effects on bacterial growth, while black pepper and red pepper extracts were found to be less inhibitory.

**TABLE 2:** The effects of plant extracts on microorganism growth inoculated with TDB (log CFU/mL).

Extracts	Con (%)	*E. faecalis*	*E. coli*	*K. pneumoniae*	*P. aeruginosa*	*S. aureus*
Control	0.00	9.23 ± 0.43 a,*	7.90 ± 0.30 a	8.19 ± 0.31 a	9.27 ± 0.34 a	8.17 ± 0.33 a
Oregano	0.02	7.25 ± 0.31 g	6.39 ± 0.36 g	7.55 ± 0.37 d	8.88 ± 0.48 d	7.53 ± 0.39 d
	0.10	2.45 ± 0.04 i	2.76 ± 0.09 i	4.27 ± 0.24 g	4.88 ± 0.43 i	2.15 ± 0.11 h
	0.50	0.00 ± 0.00 j	0.00 ± 0.00 j	0.00 ± 0.00 j	0.00 ± 0.00 j	0.00 ± 0.00 j
Black Pepper	0.02	8.73 ± 0.44 b	7.56 ± 0.37 b	7.92 ± 0.43 bc	9.14 ± 0.53 ab	7.64 ± 0.39 cd
	0.10	8.33 ± 0.32 c	7.04 ± 0.32 e	7.53 ± 0.47 d	8.50 ± 0.42 e	7.15 ± 0.45 e
	0.50	7.95 ± 0.32 e	6.74 ± 0.23 f	7.03 ± 0.36 e	8.03 ± 0.38 g	6.83 ± 0.33 f
Red Pepper	0.02	8.81 ± 0.49 b	7.64 ± 0.31 b	7.96 ± 0.34 b	9.08 ± 0.51 bc	7.71 ± 0.37 c
	0.10	8.42 ± 0.33 c	7.15 ± 0.45 d	7.57 ± 0.42 d	8.28 ± 0.38 f	7.24 ± 0.41 e
	0.50	8.22 ± 0.43 d	7.04 ± 0.29 e	7.52 ± 0.38 d	8.05 ± 0.44 g	6.93 ± 0.32 f
Cumin	0.02	7.36 ± 0.32 f	7.39 ± 0.31 c	7.84 ± 0.37 c	8.92 ± 0.49 cd	7.86 ± 0.36 b
	0.10	3.76 ± 0.05 h	3.48 ± 0.16 h	5.10 ± 0.31 f	5.88 ± 0.31 h	4.07 ± 0.13 g
	0.50	0.00 ± 0.00 j	0.00 ± 0.00 j	0.00 ± 0.00 j	0.00 ± 0.00 j	0.00 ± 0.00 j

* The numbers in the table represent the mean and standard error. Means with different letters are significantly different (*p* ≤ 0.05).

The effectiveness of plant active compounds on microorganisms can vary depending on the location where the plant is collected, the drying methods, and the extraction techniques. In a study, it was determined that cumin, red and black pepper showed low antimicrobial activity inhibition against *S. aureus* , *E. faecalis* , *K. pneumoniae* , and *P. aeruginosa* in TDB (Kuley et al. 2024). In the same study, it was shown that extracts obtained by the Soxhlet extraction method at 1% concentration effectively inhibited bacterial growth and reduced the bacterial count below 1.0 log CFU/mL (Kuley et al. 2024).

Another study (Mostafa et al. 2018) examined the antimicrobial properties of cumin extract and found limited activity against some bacterial strains. The findings suggested that the efficacy of cumin extract may be affected by its chemical composition and the targeted bacterial strains. In contrast, one study found that *Cuminum cyminum* extract exhibited potent antibacterial effects against Gram‐negative and Gram‐positive bacteria. This study showed that *C. cyminum* extract had a potent activity against uropathogenic isolates compared to amoxicillin, but its activity was not greater than that of other antibiotics (Saee et al. 2016). There are also other studies that highlight the effect of extraction solvents and drying methods on the antimicrobial properties of extracts. These studies have shown that drying techniques play an important role in affecting the stability of bioactive compounds, while solvents have a significant effect on the solubility and extraction of active compounds responsible for antimicrobial activity (Mohammad Salamatullah et al. 2022; Pundir and Jain 2010). Furthermore, antimicrobial tests indicate that extracts obtained from plant materials of the same genus and species from different locations may exhibit different bioactivity. In this context, cumin from Bulgaria was found to exhibit high activity against molds and bacteria (Jirovetz et al. 2005).

### 3.3 The Ability of Microorganisms Inoculated Into TDB on BAs Formation

**FIGURE 2:**
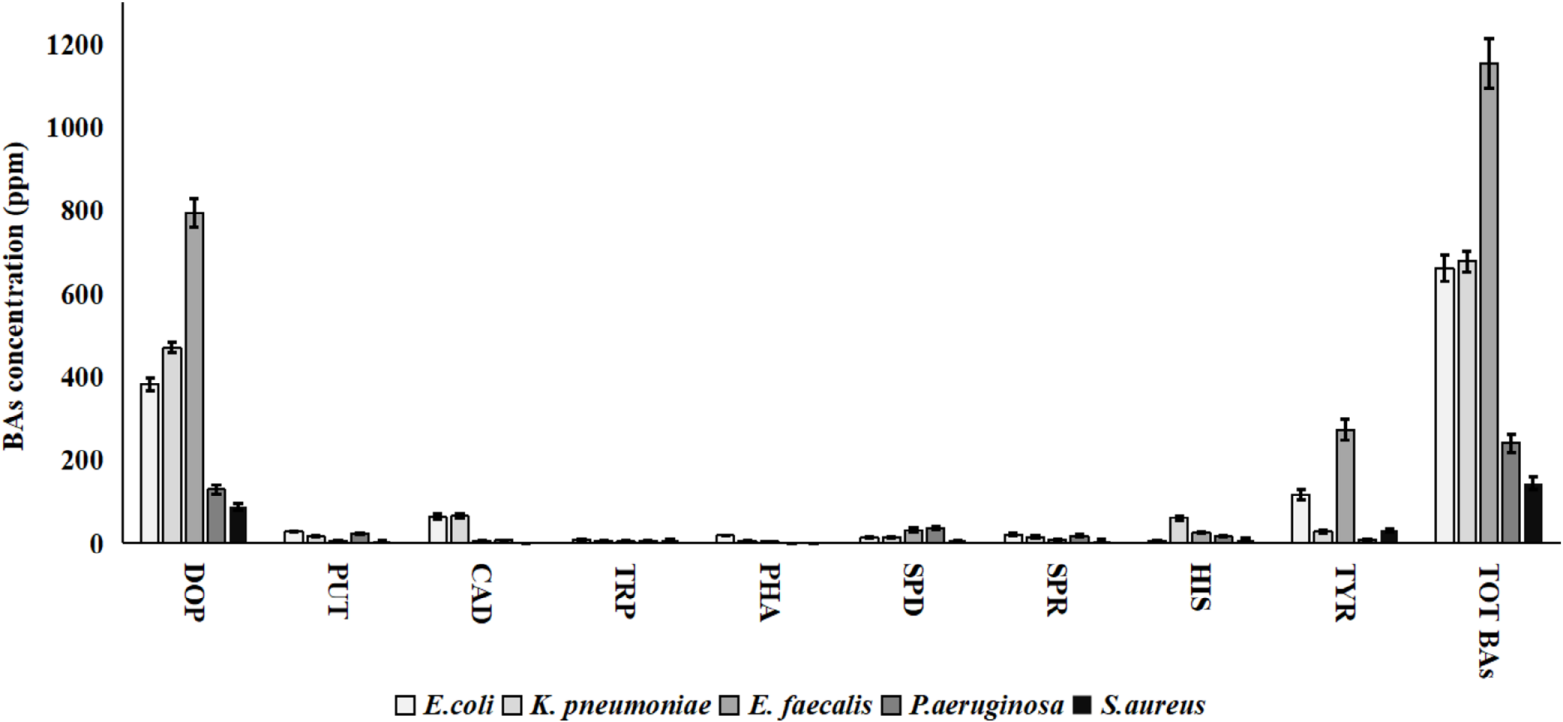
Biogenic amine production by bacteria in TDB.

No detectable biogenic amines were observed in the uninoculated negative control throughout the study, confirming that the TDB itself did not contribute to biogenic amine formation under the experimental conditions. Figure 2 shows the effects of different bacterial strains on BAs formation in TDB. According to the data, *E. faecalis* was found to be the microorganism causing the highest level of BAs formation with 794.90 mg/L DOP, 273.21 mg/L TYR, and 1153.88 mg/L total BA production. *K. pneumoniae* ranked second with 470.84 mg/L DOP and 677.78 mg/L total BA formation. *E. coli* , which ranked third in total BAs formation, caused high levels of CAD production (63.83 mg/L) compared to other strains, particularly with 382.33 mg/L DOP and 117.22 mg/L TYR. Among the microorganisms examined, *P. aeruginosa* was found to contribute to relatively lower levels of BA production overall but was responsible for the highest SPD production. In contrast, *S. aureus* was the species that showed the least contribution to total BA production in the study.

*E. coli* has been shown to produce high amounts of BAs, including high levels of PUT, CAD, PHEN, and SPR. These findings are consistent with existing literature suggesting that *E. coli* may be one of the most prevalent strains when it comes to BAs production (Özogul et al. 2018). One study evaluated the BA production capacity of specific microorganisms in TDB and showed that *E. coli* , *S. aureus* , *E. faecalis* , *K. pneumoniae* , and *P. aeruginosa* produced very low amounts of HIS (< 4 mg/L) and low to moderate levels of SPD, SPR, and PHEN. Interestingly, *E. coli* was observed to produce more TYR than other Gram‐negative bacteria. Among the Gram‐negative bacteria, *K. pneumoniae* produced the least amounts of histamine and tyrosine (Özogul et al. 2018).

In the research by Özogul et al. (2015), *E. faecalis* produced PUT at a relatively low concentration (0.89 mg/L) in the TDB, while *S. aureus* produced the highest PUT concentration (139 mg/L). In the present study, *E. faecalis* produced PUT at a significantly higher concentration (7.38 mg/L), while *S. aureus* produced the lowest PUT concentration (0.85 mg/L). These differences are likely due to the use of different bacterial strains in each study.

In a study by Engesser (1990), *E. faecalis* has been reported to produce high levels of TYR (526 mg/L) in the TDB. In the same study, *E. faecalis* was identified as a good TYR producer, while *K. pneumoniae* was classified as a moderate TYR producer. Conversely, Pseudomonas species obtained from cold‐smoked fish in vacuum packs in Portugal were identified as high HIS producers (da Silva et al. 2002). In a study by Özogul and Özogul (2007a), different strains of *K. pneumoniae* were reported to produce varying amounts of HIS. In this regard, *K. pneumoniae* strain (673) produced > 3400 mg/L HIS in HDB, while *K. pneumoniae* (8152), *E. faecalis* , and *K. pneumoniae* (2122) produced < 10 mg/L HIS. In terms of PUT and CAD production, the tested strains showed low to moderate production in TDB.

In the studies by Kuley and Özogul (2011), the total BAs production for *E. faecalis* in TDB was reported to be 884 mg/L. These results were consistent with our findings, where *E. faecalis* produced high levels of BAs. In another study, *E. faecalis* is described as the main Gram‐positive BAs producer, while it is emphasized that *S. aureus* has no ability to produce SPD, TRP, or HIS (Özogul et al. 2018). These reported data were consistent with the findings in the study, where *E. faecalis* has a high amino acid production capacity, while *S. aureus* has a very low amino acid production capacity in TDB.

### 3.4 Effects of Plant Extracts on BAs Formed by Microorganisms in TDB

In our study, the effects of various concentrations of plant extracts (oregano, black pepper, red pepper, and cumin) on BAs produced by *E. coli* , *S. aureus* , *E. faecalis* , *P. aeruginosa* , and *K. pneumoniae* were investigated. The study revealed notable inhibitory and stimulatory effects of these extracts across different bacterial strains, as detailed in Tables 3, 4, 5, 6, 7.

**TABLE 3:** The impact of plant extracts (mg/L) on the biogenic amines produced by *E. coli* in TDB.

Extracts	Con. (%)	DOP	PUT	CAD	TRP	PHEN	SPD	SPR	HIS	TYR	TOT BAs
Control	0.00	382.33 ± 5.74 a	28.28 ± 1.39 c	63.83 ± 6.4 e	8.77 ± 0.95 e	18.92 ± 1.74 d	15.31 ± 1.57 e	21.63 ± 2.16 c	6.33 ± 0.51 g	117.22 ± 7.43 a	662.66 ± 22.79 a
Oregano	0.02	356.24 ± 1.93 b	17.32 ± 0.84 de	54.64 ± 3.99 e	5.39 ± 0.16 f	11.35 ± 0.25 g	9.52 ± 1.12 h	22.69 ± 1.93 c	0.00 ± 0 h	26.95 ± 1.53 d	504.12 ± 14.61 c
	0.10	191.32 ± 1.58 ef	1.43 ± 0.02 f	14.24 ± 0.17 f	1.55 ± 0.04 g	2.31 ± 0.23 ij	2.33 ± 0.12 i	26.68 ± 2.43 b	36.82 ± 1.11 c	66.43 ± 0.14 b	343.15 ± 13.69 i
	0.50	144.68 ± 1.97 hi	1.03 ± 0.02 f	8.01 ± 0.11 fg	2.69 ± 0.06 g	1.92 ± 0.05 j	2.81 ± 0.08 i	48.46 ± 2.51 a	57.04 ± 3.5 b	29.08 ± 1.26 d	295.75 ± 14.57 j
Black pepper	0.02	281.17 ± 2.95 c	38.11 ± 1.37 b	117.07 ± 0.92 b	22.13 ± 0.22 a	34.56 ± 0.37 a	19.78 ± 1.19 b	21.65 ± 1.2 c	11.55 ± 0.85 f	42.12 ± 3.8 c	588.20 ± 17.71 b
	0.10	187.44 ± 1.29 fg	13.27 ± 0.11 de	103.75 ± 0.19 c	19.34 ± 1.19 b	28.64 ± 2.42 b	18.66 ± 1.25 c	16.24 ± 0.86 e	22.87 ± 1.08 e	14.96 ± 1.29 e	425.20 ± 13.51 fg
	0.50	159.71 ± 2.87 h	12.41 ± 0.19 de	95.72 ± 0.67 c	15.10 ± 1.17 c	22.90 ± 1.55 c	22.17 ± 2.13 a	19.43 ± 1.22 d	80.76 ± 4.19 a	35.32 ± 3.72 cd	463.55 ± 16.26 de
Red pepper	0.02	341.57 ± 3.57 b	10.12 ± 0.16 e	58.06 ± 1.78 e	8.66 ± 0.38 e	16.33 ± 0.34 e	17.29 ± 1.19 d	15.45 ± 1.13 e	12.72 ± 0.12 f	7.07 ± 0.58 ef	487.32 ± 12.33 cd
	0.10	278.69 ± 2.15 c	14.19 ± 1.21 de	57.50 ± 4.53 e	7.65 ± 4.18 e	13.77 ± 1.11 f	13.34 ± 1.23 f	13.69 ± 0.89 f	36.26 ± 0.32 c	8.69 ± 0.6 e	443.81 ± 13.46 ef
	0.50	209.05 ± 2.02 d	17.60 ± 1.26 d	79.55 ± 5.23 d	10.43 ± 4.19 d	18.11 ± 1.55 d	19.57 ± 1.25 bc	14.43 ± 1.15 f	34.94 ± 2.08 c	11.56 ± 0.54 e	415.26 ± 15.34 g
Cumin	0.02	138.71 ± 1.56 i	47.46 ± 8.39 a	128.62 ± 6.17 a	5.61 ± 0.18 f	9.66 ± 0.15 h	10.89 ± 1.3 g	7.96 ± 0.4 g	20.97 ± 2.38 e	10.67 ± 1.5 e	380.57 ± 19.03 h
	0.10	206.50 ± 4.77 de	1.65 ± 0.03 f	1.21 ± 0.53 g	0.00 ± 0 h	2.33 ± 0.29 ij	0.00 ± 0 j	5.50 ± 0.31 h	30.14 ± 1.91 d	0.00 ± 0 f	247.36 ± 15.18 k
	0.50	175.08 ± 6.84 g	1.23 ± 0.25 f	1.61 ± 0.02 g	0.00 ± 0 h	3.54 ± 0.37 i	0.00 ± 0 j	3.51 ± 0.06 i	82.15 ± 3.5 a	0.00 ± 0 f	267.14 ± 16.42 k

*Note:* The numbers in the table represent means and standard errors. Significant differences between means are indicated by different letters (*p* ≤ 0.05).

Abbreviations: CAD, cadaverine; Con, concentration; DOP, dopamine; HIS, histamine; PHEN, phenylethylamine; PUT, putrescine; SPD, spermidine; SPR, spermine; TOT BAs, total biogenic amines; TRP, tryptamine; TYR, tyramine.

In the control group of *E. coli* (Table 3), significant levels of BAs were observed, with DOP at 382.33 mg/L, TYR at 117.22 mg/L, CAD at 63.84 mg/L, and PUT at 28.28 mg/L, resulting in a total BAs concentration of 662.66 mg/L. In terms of BAs inhibition, oregano extract exhibited a significant and strong inhibitory effect, particularly at concentrations of 0.10% and 0.50%, inhibiting the formation of DOP, CAD, and TYR. In contrast, black pepper extract, particularly at a concentration of 0.50%, significantly stimulated BAs production, increasing the HIS level to 80.76 mg/L and reaching a total BAs concentration of 463.55 mg/L. Red pepper extract demonstrated a mixed effect at low concentrations, inhibiting PUT, CAD, TRP, and PHEN, while stimulating HIS production at all concentrations. Cumin extract showed a significant inhibitory effect at all concentrations, particularly reducing TYR, leading to the lowest total BAs concentration of 267.14 mg/L at 0.50%.

For *S. aureus* , moderate BAs levels were observed in the control group, especially for DOP (88.09 mg/L), TYR (30.39 mg/L), and HIS (9.48 mg/L), with a total BAs concentration of 144.75 mg/L (Table 4). Oregano extract, particularly at a concentration of 0.10%, significantly inhibited amino acid formation, reducing the total BAs level to 82.20 mg/L. Black pepper extract showed a mixed effect, with an inhibitory action at low concentrations and a stimulating effect on TRP at 0.50%. Red pepper extract stimulated BAs formation at all concentrations, especially increasing CAD and SPD. Cumin extract exhibited a significant inhibitory effect by reducing DOP, HIS, and TYR.

**TABLE 4:** The impact of plant extracts (mg/L) on the biogenic amines produced by *S. aureus* in TDB.

Extracts	Con. (%)	DOP	PUT	CAD	TRP	PHEN	SPD	SPR	HIS	TYR	TOT BAs
Control	0.00	88.09 ± 3.42 bc	0.85 ± 0.05 c	0.000 ± 0 b	4.34 ± 0.28 cd	1.20 ± 0.10 a	3.29 ± 0.27 e	7.74 ± 3.8 bc	9.48 ± 0.56 cd	30.93 ± 3.82 a	144.75 ± 6.61 cde
Oregano	0.02	84.33 ± 1.93 c	1.60 ± 0.06 c	0.000 ± 0 b	2.71 ± 0.18 defg	0.00 ± 0.00 b	2.39 ± 0.13 e	11.51 ± 0.02 bc	6.97 ± 0.46 de	5.84 ± 0.58 ef	115.37 ± 9.21 def
	0.10	38.72 ± 0.92 f	0.00 ± 0.00 c	0.000 ± 0 b	2.97 ± 0.22 cdef	0.00 ± 0.00 b	4.12 ± 0.35 e	24.41 ± 2.04 b	6.00 ± 0.43 e	5.96 ± 0.51 ef	82.20 ± 4.57 ef
	0.50	16.68 ± 0.34 g	0.00 ± 0.00 c	0.000 ± 0 b	1.74 ± 0.11 fgh	0.00 ± 0.00 b	2.68 ± 0.28 e	53.05 ± 4.06 a	4.69 ± 0.37 e	14.83 ± 1.65 c	93.70 ± 5.69 ef
Black pepper	0.02	74.17 ± 0.61 d	0.00 ± 0.00 c	1.511 ± 0.18 b	4.50 ± 0.21 c	0.00 ± 0.00 b	11.32 ± 1.32 b	5.60 ± 0.39 bc	97.39 ± 5.48 a	23.83 ± 1.52 b	218.34 ± 13.46 ab
	0.10	8.17 ± 0.41 h	0.00 ± 0.00 c	2.711 ± 0.13 b	14.50 ± 0.7 b	0.00 ± 0.00 b	4.32 ± 0.32 e	13.16 ± 1.77 bc	18.35 ± 1.76 b	11.24 ± 0.92 cde	72.47 ± 3.30 f
	0.50	3.88 ± 0.81 h	14.25 ± 1.32 b	4.448 ± 0.38 b	24.73 ± 1.2 a	0.00 ± 0.00 b	2.46 ± 0.18 e	7.22 ± 0.29 bc	12.33 ± 1.39 c	14.09 ± 0.14 cd	83.43 ± 6.17 ef
Red pepper	0.02	93.87 ± 4.17 ab	0.00 ± 0.00 c	129.779 ± 9.66 a	1.28 ± 0.1 gh	0.00 ± 0.00 b	11.36 ± 0.89 b	12.05 ± 1.11 bc	0.00 ± 0.00 f	6.76 ± 0.46 ef	255.12 ± 12.95 a
	0.10	47.61 ± 2.73 e	0.00 ± 0.00 c	124.244 ± 11.63 a	0.22 ± 0 h	0.00 ± 0.00 b	9.27 ± 0.79 c	6.71 ± 0.48 bc	0.00 ± 0.00 f	4.11 ± 0.38 f	192.18 ± 11.34 abc
	0.50	54.46 ± 2.52 e	0.00 ± 0.00 c	91.014 ± 5.14 a	1.48 ± 0.07 fgh	0.00 ± 0.00 b	13.36 ± 1.29 a	10.57 ± 0.85 bc	0.00 ± 0.00 f	7.31 ± 0.62 ef	178.22 ± 12.76 bcd
Cumin	0.02	97.09 ± 6.95 a	0.00 ± 0.00 c	0.00 ± 0.00 b	3.51 ± 0.21 cde	0.00 ± 0.00 b	6.61 ± 0.21 d	5.54 ± 0.43 bc	0.00 ± 0.00 f	4.13 ± 0.21 f	116.90 ± 7.03 def
	0.10	70.63 ± 4.15 d	36.86 ± 3.60 a	0.00 ± 0.00 b	1.50 ± 0.09 fgh	0.00 ± 0.00 b	6.97 ± 0.58 d	3.71 ± 0.23 c	0.00 ± 0.00 f	4.40 ± 0.30 f	124.10 ± 7.45 def
	0.50	33.97 ± 2.11 f	3.24 ± 0.16 c	0.00 ± 0.00 b	2.29 ± 0.12 efg	0.00 ± 0.00 b	9.19 ± 0.81 c	3.19 ± 0.39 c	5.67 ± 0.48 e	8.44 ± 0.77 def	66.02 ± 5.71 f

*Note:* The numbers in the table represent means and standard errors. Significant differences between means are indicated by different letters (p ≤ 0.05).

Abbreviations: CAD, cadaverine; Con, concentration; DOP, dopamine; HIS, histamine; PHEN, phenylethylamine; PUT, putrescine; SPD, spermidine; SPR, spermine; TOT BAs, total biogenic amines; TRP, tryptamine; TYR, tyramine.

For *E. faecalis* , the control group exhibited high BAs levels, particularly DOP (794.90 mg/L), TYR (273.21 mg/L), and HIS (25.79 mg/L), with a total BAs concentration of 1153.88 mg/L (Table 5). Oregano extract significantly inhibited amino acid formation at all concentrations, especially at 0.50%, reducing the total BAs to 124.14 mg/L. Black pepper extract inhibited the production of DOP, HIS, and TYR at all concentrations, showing a significant inhibitory effect. Red pepper extract stimulated BAs formation, but significantly inhibited PUT, HIS, and TYR production. Cumin extract exhibited a significant inhibitory effect at concentrations of 0.10% and 0.50%, reducing DOP, HIS, TYR, and total BAs.

**TABLE 5:** The impact of plant extracts (mg/L) on the biogenic amines produced by *E. faecalis* in TDB.

Extracts	Con. (%)	DOP	PUT	CAD	TRP	PHEN	SPD	SPR	HIS	TYR	TOT BAs
Control	0.00	794.90 ± 30.6 a	7.38 ± 0.34 b	2.34 ± 0.17 def	6.73 ± 0.37 b	1.24 ± 0.14 e	32.48 ± 2.7 a	9.78 ± 0.84 cd	25.79 ± 2.01 a	273.21 ± 20.91 b	1153.88 ± 12.06 a
Oregano	0.02	164.72 ± 7.89 d	1.68 ± 0.17 de	0.86 ± 0.04 efg	2.56 ± 0.16 e	1.13 ± 0.05 e	0.93 ± 0.01 fg	6.34 ± 0.54 e	8.96 ± 0.47 f	296.65 ± 12.4 b	483.87 ± 9.27 c
	0.10	193.02 ± 12.22 d	1.70 ± 0.11 de	1.05 g ± 0.09 ef	2.81 ± 0.17 de	1.10 ± 0.11 e	1.79 ± 0.12 def	12.45 ± 1.34 b	17.88 ± 0.12 cd	12.69 ± 0.64 e	244.52 ± 16.80 f
	0.50	73.08 ± 5.75 f	0.93 ± 0.05 e	0.61 ± 0.04 fg	2.59 ± 1.04 e	2.48 ± 0.24 b	0.00 ± 0.00 g	10.37 ± 0.61 c	16.93 ± 1.29 d	17.12 ± 1.69 e	124.14 ± 7.31 h
Black pepper	0.02	95.18 ± 7.63 ef	1.04 ± 0.08 e	0.00 ± 0.00 g	3.92 ± 0.16 cd	2.32 ± 0.13 bc	1.96 ± 0.11 def	5.34 ± 0.19 e	10.97 ± 1.63 e	123.38 ± 11.27 c	244.14 ± 11.19 f
	0.10	102.41 ± 9.63 ef	2.82 ± 0.11 cd	0.00 ± 0.00 g	3.87 ± 0.14 cd	1.73 ± 0.11 cde	1.57 ± 0.13 ef	8.22 ± 0.63 d	18.89 ± 1.36 bc	149.18 ± 9.24 c	288.72 ± 15.11 e
	0.50	110.36 ± 9.04 e	2.83 ± 0.15 cd	2.72 ± 0.07 de	1.88 ± 0.58 ef	1.61 ± 0.12 de	1.78 ± 0.14 def	9.15 ± 0.53 cd	19.93 ± 1.53 b	136.56 ± 12.81 c	286.86 ± 13.88 e
Red pepper	0.02	226.39 ± 16.45 c	1.51 ± 0.08 de	13.54 ± 0.28 b	13.39 ± 1.01 a	3.57 ± 0.2 a	2.82 ± 0.17 cd	15.01 ± 1.22 a	0.00 ± 0.00 h	64.87 ± 4.16 d	341.13 ± 18.51 d
	0.10	188.15 ± 13.19 d	1.91 ± 0.13 de	4.98 ± 0.25 c	0.92 ± 0.06 fg	2.49 ± 0.18 b	2.71 ± 0.21 cde	9.27 ± 0.81 cd	0.00 ± 0.00 h	69.08 ± 3.55 d	279.54 ± 12.97 e
	0.50	120.53 ± 8.82 e	1.15 ± 0.08 e	17.62 ± 0.98 a	4.04 ± 0.19 c	2.21 ± 0.17 bcd	3.83 ± 0.12 c	10.38 ± 1.48 c	0.00 ± 0.00 h	7.21 ± 0.38 e	166.99 ± 14.64 g
Cumin	0.02	539.28 ± 21.46 b	4.04 ± 0.34 c	3.61 ± 0.18 cd	5.61 ± 0.37 b	0.00 ± 0.00 f	22.27 ± 2.24 b	5.34 ± 0.24 e	0.00 ± 0.00 h	385.22 ± 22.4 a	965.41 ± 24.48 b
	0.10	110.05 ± 9.29 e	6.56 ± 0.49 b	1.33 ± 0.06 efg	1.72 ± 0.05 ef	0.00 ± 0.00 f	3.83 ± 0.15 c	3.71 ± 0.20 f	0.00 ± 0.00 h	25.93 ± 2.64 e	153.15 ± 2.22 g
	0.50	102.37 ± 7.69 ef	21.57 ± 1.26 a	0.00 ± 0.00 g	0.00 ± 0.00 g	0.00 ± 0.00 f	0.00 ± 0.00 g	2.63 ± 0.21 f	4.60 ± 0.20 g	23.22 ± 2.30 e	154.41 ± 0.86 g

*Note:* The numbers in the table represent means and standard errors. Significant differences between means are indicated by different letters (p ≤ 0.05).

Abbreviations: CAD, cadaverine; Con, concentration; DOP, dopamine; HIS, histamine; PHEN, phenylethylamine; PUT, putrescine; SPD, spermidine; SPR, spermine; TOT BAs, total biogenic amines; TRP, tryptamine; TYR, tyramine.

For *P. aeruginosa* , significant BAs production was observed in the control group, especially DOP (129.81 mg/L), SPD (36.43 mg/L), and PUT (23.68 mg/L), with a total BAs concentration of 242.20 mg/L (Table 6). Oregano extract, particularly at 0.50%, significantly inhibited amino acid formation, reducing the total BAs concentration to 135.34 mg/L. Black pepper extract showed an inhibitory effect at low concentrations and increased HIS (153.47 mg/L) and total BAs (277.39 mg/L) at 0.50%. Red pepper extract displayed various effects, particularly stimulating TRP production. Cumin extract demonstrated a significant inhibitory effect by reducing DOP, PUT, and TYR.

**TABLE 6:** The impact of plant extracts (mg/L) on the biogenic amines produced by *P. aeruginosa* in TDB.

Extracts	Con. (%)	DOP	PUT	CAD	TRP	PHEN	SPD	SPR	HIS	TYR	TOT BAs
Control	0.00	129.81 ± 11.69 a	23.68 ± 1.38 cd	3.85 ± 0.47 cd	1.63 ± 0.13 g	1.33 ± 0.33 a	36.43 ± 2.52 a	18.46 ± 1.49 d	16.55 ± 1.69 c	10.43 ± 1.46 d	242.20 ± 12.22 ab
Oregano	0.02	85.95 ± 4.64 bc	2.12 ± 0.17 f	5.57 ± 0.44 c	1.64 ± 0.64 g	0.00 ± 0.00 b	24.75 ± 1.45 c	17.25 ± 1.07 d	6.43 ± 0.64 d	16.59 ± 0.96 c	160.33 ± 13.66 ef
	0.10	76.57 ± 3.36 cd	1.28 ± 0.12 f	1.14 ± 0.09 cd	4.13 ± 0.36 f	0.00 ± 0.00 b	9.36 ± 0.2 g	31.80 ± 2.03 b	24.16 ± 2.28 b	35.87 ± 2.59 a	184.36 ± 13.38 de
	0.50	43.08 ± 3.13 f	1.78 ± 0.07 f	0.00 ± 0.00 d	1.88 ± 0.13 g	0.00 ± 0.00 b	13.24 ± 1.33 f	47.17 ± 3.05 a	5.82 ± 0.67 d	22.34 ± 2.01 b	135.34 ± 10.62 fg
Black pepper	0.02	53.45 ± 3.84 ef	23.68 ± 2.1 cd	3.19 ± 0.26 cd	1.38 ± 0.14 g	0.00 ± 0.00 b	16.31 ± 1.36 e	12.15 ± 1.52 e	15.08 ± 1.04 c	8.21 ± 0.55 de	133.47 ± 11.62 fg
	0.10	40.98 ± 2.11 f	20.26 ± 1.35 cd	2.04 ± 0.14 cd	7.13 ± 0.41 e	0.00 ± 0.00 b	3.13 ± 0.15 h	10.24 ± 1.4 e	16.96 ± 1.22 c	7.22 ± 0.36 def	107.99 ± 9.13 g
	0.50	50.67 ± 3.4 ef	26.37 ± 2.29 c	2.99 ± 0.17 cd	13.27 ± 1.4 d	0.00 ± 0.00 b	4.15 ± 0.12 h	16.67 ± 1.59 d	153.47 ± 13.73 a	9.76 ± 1.08 d	277.39 ± 12.66 a
Red pepper	0.02	94.45 ± 5.35 b	34.71 ± 2.32 b	0.00 ± 0.00 d	36.03 ± 2.35 b	0.00 ± 0.00 b	13.12 ± 1.21 f	11.01 ± 1.14 e	5.52 ± 0.25 d	7.20 ± 0.62 def	202.06 ± 17.68 cd
	0.10	87.07 ± 5.65 bc	40.68 ± 3.68 ab	0.00 ± 0.00 d	24.44 ± 1.65 c	0.00 ± 0.00 b	18.23 ± 1.18 e	16.82 ± 1.01 d	16.22 ± 1.2 c	7.63 ± 0.71 def	211.11d ± 13.1 bc
	0.50	73.08 ± 4.13 cd	44.32 ± 2.32 a	0.00 ± 0.00 d	44.15 ± 3.13 a	0.00 ± 0.00 b	28.50 ± 2.17 b	22.48 ± 2.07 c	3.62 ± 0.28 d	8.67 ± 0.59 d	224.86 ± 16.49 bc
Cumin	0.02	91.96 ± 6.65 b	10.28 ± 1.10 e	24.23 ± 1.43 b	1.44 ± 0.15 g	0.00 ± 0.00 b	20.96 ± 2.08 d	3.05 ± 2.84 f	15.82 ± 1.68 c	3.86 ± 0.22 efg	156.30 ± 13.47 ef
	0.10	64.09 ± 4.25 de	20.00 ± 1.64 cd	38.35 ± 3.69 a	0.77 ± 0.05 g	0.00 ± 0.00 b	8.14 ± 0.43 g	4.69 ± 3.07 f	12.32 ± 0.82 c	2.38 ± 0.32 g	140.08 ± 11.58 fg
	0.50	53.71 ± 3.47 ef	16.78 ± 1.12 de	0.00 ± 0.00 d	0.00 ± 0.00 g	0.00 ± 0.00 b	16.38 ± 1.32 e	3.13 ± 0.24 f	26.44 ± 1.74 b	3.08 ± 0.24 fg	110.59 ± 9.10 g

*Note:* The numbers in the table represent means and standard errors. Significant differences between means are indicated by different letters (p ≤ 0.05).

Abbreviations: CAD, cadaverine; Con, concentration; DOP, dopamine; HIS, histamine; PHEN, phenylethylamine; PUT, putrescine; SPD, spermidine; SPR, spermine; TOT BAs, total biogenic amines; TRP, tryptamine; TYR, tyramine.

For *K. pneumoniae* , significant levels of BAs were observed, particularly DOP (470.84 mg/L), HIS (61.34 mg/L), and CAD (66.05 mg/L), contributing to a total BAs concentration of 677.78 mg/L (Table 7). Oregano extract significantly inhibited amino acid formation, particularly by reducing DOP and CAD. Black pepper extract exhibited a pronounced ability to completely inhibit HIS production. Red pepper extract primarily stimulated CAD production. Cumin extract demonstrated a high inhibitory effect, particularly by reducing DOP and HIS. These results highlight the varying effects of these extracts on BAs formation by different bacterial strains and contribute to the quality and safety of foods, aiding in the protection of public health through the control of BAs formation in food products. The oregano extract, which was found to be the most effective in BAs inhibition in this study, has also been emphasized in many studies. An experiment on the impact of oregano extract on BAs formation in sardine slices, this study indicated that oregano extract significantly inhibited BAs formation, with BAs content being detected to be approximately 10 times lower than in control samples on Day 35 of storage (Křížek et al. 2018).

**TABLE 7:** The impact of plant extracts (mg/L) on the biogenic amines produced by *K. pneumoniae* in TDB.

Extracts	Con. (%)	DOP	PUT	CAD	TRP	PHEN	SPD	SPR	HIS	TYR	TOT BAs
Control	0.00	470.84 ± 26.73 a	16.90 ± 0.94 cd	66.05 ± 3.04 f	1.98 ± 0.09 a	2.43 ± 0.16 a	14.63 ± 1.22 a	15.12 ± 2.55 c	61.34 ± 4.52 a	28.46 ± 2.37 c	677.78 ± 22.56 a
Oregano	0.02	258.04 ± 11.9 d	13.85 ± 1.31 de	165.98 ± 10.86 a	0.00 ± 0.00 c	0.00 ± 0.00 b	0.00 ± 0.00 d	14.67 ± 1.18 c	8.82 ± 0.82 b	8.99 ± 0.41 fg	470.37 ± 26.32 c
	0.10	134.64 ± 11.79 g	10.68 ± 0.96 efg	13.51 ± 10.16 h	0.00 ± 0.00 c	0.00 ± 0.00 b	0.00 ± 0.00 d	28.95 ± 2.16 b	53.29 ± 4.43 a	88.47 ± 5.33 b	329.57 ± 12.3 g
	0.50	54.61 ± 4.44 i	33.56 ± 2.78 a	13.94 ± 10.22 h	0.00 ± 0.00 c	0.00 ± 0.00 b	0.00 ± 0.00 d	44.67 ± 2.57 a	57.47 ± 3.7 a	103.83 ± 10.57 a	308.09 ± 13.57 h
Black pepper	0.02	351.33 ± 15.86 b	8.72 ± 0.84 fg	85.24 ± 7.74 e	0.00 ± 0.00 c	0.00 ± 0.00 b	6.89 ± 0.64 b	6.79 ± 0.54 de	0.00 ± 0.00 c	12.39 ± 0.93	471.38 ± 13.32 c
	0.10	216.48 ± 12.43 e	7.19 ± 0.64 g	104.89 ± 12.25 d	0.00 ± 0.00 c	0.00 ± 0.00 b	7.49 ± 0.41 b	6.87 ± 0.41 de	0.00 ± 0.00 c	13.33 ± 0.47 d	356.26 ± 14.14 f
	0.50	272.87 ± 10.65 c	10.10 ± 0.84 fg	89.33 ± 6.5 e	0.00 ± 0.00 c	0.00 ± 0.00 b	14.51 ± 1.2 a	13.57 ± 1.25 c	0.00 ± 0.00 c	11.38 ± 1.19 def	411.78 ± 16.66 d
Red pepper	0.02	351.33 ± 15.86 b	22.74 ± 1.22 b	149.00 ± 12.33 b	0.00 ± 0.00 c	0.00 ± 0.00 b	7.75 ± 0.52 b	16.37 ± 1.03 c	56.81 ± 5.06 a	10.15 ± 0.87 ef	614.17 ± 29.43 b
	0.10	216.48 ± 12.43 e	18.16 ± 1.4 c	129.86 ± 8.54 c	0.00 ± 0.00 c	0.00 ± 0.00 b	1.28 ± 0.08 d	11.94 ± 1.01 cd	7.14 ± 0.42 bc	6.15 ± 0.22 h	391.03 ± 25.44 e
	0.50	272.87 ± 10.65 c	11.92 ± 0.92 ef	83.42 ± 6.34 e	1.53 ± 0.05 b	0.00 ± 0.00 b	0.00 ± 0.00 d	6.45 ± 0.55 de	0.00 ± 0.00 c	7.10 ± 0.48 gh	383.30 ± 21.64 e
Cumin	0.02	277.93 ± 18.95 c	9.32 ± 0.92 fg	91.30 ± 8.01 de	0.00 ± 0.00 c	0.00 ± 0.00 b	13.26 ± 1.29 a	4.11 ± 0.29 e	11.91 ± 1.08 b	6.44 ± 0.3 h	414.31 ± 24.38
	0.10	167.63 ± 15.89 f	0.00 ± 0.00 h	38.39 ± 2.58 g	0.00 ± 0.00 c	0.00 ± 0.00 b	3.81 ± 0.1 c	3.76 ± 0.29 e	9.32 ± 0.32 b	5.41 ± 0.33 h	228.34 ± 16.13 i
	0.50	81.56 ± 5.52 h	0.00 ± 0.00 h	19.99 ± 1.84 h	0.00 ± 0.00 c	0.00 ± 0.00 b	0.00 ± 0.00 d	11.34 ± 0.85 cd	10.52 ± 0.84 b	12.04 ± 1.05 de	135.47 ± 12.37 j

*Note:* The numbers in the table represent means and standard errors. Significant differences between means are indicated by different letters (p ≤ 0.05).

Abbreviations: CAD, cadaverine; Con, concentration; DOP, dopamine; HIS, histamine; PHEN, phenylethylamine; PUT, putrescine; SPD, spermidine; SPR, spermine; TOT BAs, total biogenic amines; TRP, tryptamine; TYR, tyramine.

In an experiment to evaluate the impact of carvacrol on BAs formation, inhibition levels varied with carvacrol concentrations and specific bacterial strains, where the compound showed divergent effects on the tested bacteria (Dorman and Deans 2000). In another experiment, aside from TYR production, carvacrol was not active as an inhibitor of BAs production in Gram‐positive *E. faecalis* . Additionally, 1% carvacrol significantly reduced BAs production by the majority of Gram‐negative and Gram‐positive bacteria (Özogul et al. 2015). In the same study, *K. pneumoniae* and *S. aureus* production of PUT was significantly lower when treated with 0.50% and 1% carvacrol compared to the control. *E. faecalis* , though, yielded higher PUT and CAD in the presence of carvacrol. Bacterial accumulation of SPR and SPM was also suppressed when 1% carvacrol was present, except in *E. faecalis* . Bacterial TYR production was greatly suppressed when 0.50% and 1% carvacrol were added, and no suppression of TYR production was observed when 0.10% carvacrol was added to *E. faecalis* . The study demonstrated that 0.50% and 1% concentrations of carvacrol had an inhibitory effect on the biogenic amines produced by *E. coli* and *S. aureus* (Özogul et al. 2015). The results of the current study show that oregano has a strong inhibitory impact on BAs production, particularly in *E. faecalis* , with a significant reduction in the production of the majority of BAs. A study by (Burt 2004) explored the antibacterial efficacy of oregano essential oil and food application, where it was cited for its intense antimicrobial action. Furthermore, research by (Tepe et al. 2004) found that different extracts of *Origanum acutidens* of the same possessed antimicrobial, antioxidant, and antiviral activities with results parallel to the findings in the current study.

A study by Özogul et al. (2022) showed that cumin extract had the strongest antibacterial properties. Cumin extract was also effective in inhibiting foodborne pathogens and preventing formation of BAs in HDB. Our results are consistent with this study, which showed that cumin extract at different concentrations significantly reduced BAs production by the tested bacteria. Literature also reports that extracts obtained from some herbal raw materials using different solvents play a role in enhancing BAs formation when added to foods.

Therefore, the inhibitory effects observed in the present study are likely the result of multiple interacting mechanisms rather than a single mode of action. In contrast, the reduction in biogenic amine formation observed in the present study may be attributed to a combination of antimicrobial and biochemical mechanisms. The plant extracts inhibited bacterial growth, resulting in a lower population of amino acid decarboxylase‐producing microorganisms (Liu et al. 2026).

Moreover, bioactive constituents such as carvacrol and cuminaldehyde have been reported to disrupt cell membrane integrity and interfere with intracellular enzymatic processes, potentially reducing decarboxylase activity (Paul et al. 2025; Ultee et al. 2002).

The consequent decline in microbial metabolic activity further restricts the conversion of amino acid precursors, including tyrosine, histidine, ornithine, and lysine, into their respective biogenic amines (Barbieri et al. 2019; Li et al. 2023). Collectively, these effects contribute to the observed reduction in biogenic amine accumulation.

It should be noted that the complete inhibition of bacterial growth observed at the highest extract concentration (0.50%) does not necessarily imply the complete absence of biogenic amine formation. The antimicrobial compounds present in oregano and cumin extracts can disrupt bacterial cell membranes and cellular integrity, leading to growth inhibition and cell death (Burt 2004; Hyldgaard et al. 2012). However, small amounts of biogenic amines may have been formed during the early stages of incubation before complete growth suppression was achieved. Therefore, the residual biogenic amine concentrations detected in some treatments may reflect amines produced prior to the complete inhibition of bacterial growth rather than ongoing bacterial proliferation (Ercan et al. 2013; Ladero et al. 2010).

In fact, a study found that the extract of pepper obtained using diethyl ether increased BAs levels in an in vitro environment, and it was noted that this extract should be used more cautiously, particularly in foods associated with BAs poisoning, due to the potential for increased BAs production. The study also confirmed that the use of red pepper extract in food products is linked to food poisoning, as it indicated the ability of this extract to raise the concentration of HIS in food products and thus cause the formation of BAs, which may pose a threat to public health (Özogul et al. 2022).

In a Wendakoon and Sakaguchi (1992) study, the addition of pepper extract to decarboxylase broth resulted in a 10‐fold increase in HIS production by *Morganella morganii* . Furthermore, as per a study by Mah et al. (2009), red pepper extract increased the formation of PUT and HIS with no significant impact on other BAs.

It is reported that some of the extracts increase levels of BAs in decarboxylase broth by altering the pH or other properties of the decarboxylase broth. This alteration may affect the working conditions of bacterial decarboxylase enzymes, leading to variations in levels of BAs (Özogul et al. 2022). Another study about inhibitory effects of foodborne pathogen growth and BAs production by spice extracts, where extracts provided a suitable condition for enzyme activity. In the present study, the effect of 1% concentrations of red pepper, black pepper, and cumin extracts on BAs production by *S. aureus* were investigated. The findings indicated that cumin extract activated the production of most BAs, including TYR, HIS, PUT, CAD, SPM, TRP, PHEA, SPR, and DOP, while red pepper and black pepper extracts inhibited the production of TYR, DOP, PUT, and CAD but activated HIS production (Kuley et al. 2024).

Furthermore, the increase in BAs production by spice extracts can be explained by the abundance of alkaloids in spices, as it is known that alkaloid content is associated with increased BAs production (Kuley et al. 2024). Additionally, bacteria and several microorganisms have been reported to secrete endogenous and exogenous enzymes (amino acid decarboxylase enzymes) for the decarboxylation of amino acids (Gardini et al. 2016). In light of these hypotheses, the increase in BAs occurs after the destruction of microorganisms and the breakdown of cell walls, which leads to the release of enzymes into the environment. As a result, it is believed that this could lead to an increase in the synthesis or formation of BAs in decarboxylase broth.

The effects of the tested extracts on biogenic amine formation appeared to be both extract‐dependent and strain‐specific. Oregano and cumin extracts generally reduced total biogenic amine accumulation, which is consistent with their strong inhibitory effects on bacterial growth observed in Table 2. Therefore, a substantial part of the reduction in biogenic amine formation can be attributed to the decreased population of amino acid decarboxylase‐producing microorganisms (Gardini et al. 2016).

However, the responses observed with black pepper and red pepper extracts were more variable. For example, black pepper extract stimulated histamine production in *E. coli* while completely suppressing histamine formation in *K. pneumoniae* . Such strain‐specific responses may reflect differences in amino acid decarboxylase systems, regulation of decarboxylase gene expression, cellular stress responses, and susceptibility to bioactive compounds such as piperine (Gao et al. 2023; Özogul and Özogul 2007; Özogul et al. 2018).

In addition, sublethal stress caused by some plant‐derived compounds may alter microbial metabolism and selectively affect specific biogenic amine pathways (Dai et al. 2025; Gardini et al. 2016). Similar strain‐dependent variations in biogenic amine production have been reported previously and highlight the complexity of interactions between bacterial metabolism and plant bioactive compounds (Irmler et al. 2026).

## 4 Conclusions

The results showed that the oregano and cumin extracts, especially at the highest concentration (0.50%), completely inhibited the growth of all tested bacterial species. Moderate and low concentrations of oregano and cumin extracts also significantly reduced the logarithm of colony‐forming units per mL for all bacteria, particularly *E. faecalis* , demonstrating significant antibacterial activity. Furthermore, analyses of biogenic amine production in decarboxylase lysates revealed that *E. faecalis* had the highest capacity for total biogenic amines, producing 1153.88 mg/L of BAs. Meanwhile, cumin extract showed the greatest inhibitory effect on biogenic amine formation across all five bacterial species, particularly at concentrations of 0.50% and 0.10%. This resulted in the lowest total levels of BAs in *E. coli* , *S. aureus* , *P. aeruginosa* , and *K. pneumoniae* . Oregano also showed strong effects, especially at a concentration of 0.50%, but was generally less effective than cumin. These results emphasize the functional value of plant‐derived extracts, particularly cumin and oregano, as sustainable and natural inhibitors of biogenic amine production, offering a promising avenue for enhancing microbial safety and quality in food matrices.

## Author Contributions

**Mustafa Hamza Mawlood Al Bayati:** formal analysis, writing – original draft, validation, investigation, methodology. **Pinar Yerlikaya:** writing – review and editing, supervision. **Mariem Bouali:** formal analysis, validation. **Mehmet Fatih Cengiz:** writing – review and editing, supervision, project administration, methodology, investigation, conceptualization.

## Funding

This project was supported by the grants from Akdeniz University, The Scientific Research Projects Coordination Unit (FBA‐2023‐6206).

## Conflicts of Interest

The authors declare no conflicts of interest.

## Data Availability

The data that support the findings of this study are available from the corresponding author upon reasonable request.
